# CD4+ T-lymphocytes in human saccular intracranial aneurysm walls are associated with aneurysm rupture

**DOI:** 10.1093/jnen/nlaf060

**Published:** 2025-06-11

**Authors:** Nora Firtser, Eliisa Netti, Eliisa Kekäläinen, Satu Lehti, Petri T Kovanen, Mika Niemelä, Riikka Tulamo

**Affiliations:** Neurosurgery Research Group, Biomedicum 1, Helsinki, Finland; Department of Neurosurgery, Kuopio University Hospital, Kuopio, Finland; Neurosurgery Research Group, Biomedicum 1, Helsinki, Finland; Department of Neurosurgery, Helsinki University Hospital and University of Helsinki, Helsinki, Finland; Translational Immunology Research Program, University of Helsinki, Biomedicum 1, Helsinki, Finland; Gerontology Research Center, Faculty of Sport and Health Sciences, University of Jyväskylä, Jyväskylä, Finland; Wihuri Research Institute, Atherosclerosis Research Laboratory, Biomedicum 1, Helsinki, Finland; Neurosurgery Research Group, Biomedicum 1, Helsinki, Finland; Department of Neurosurgery, Helsinki University Hospital and University of Helsinki, Helsinki, Finland; Neurosurgery Research Group, Biomedicum 1, Helsinki, Finland; Department of Vascular Surgery, Helsinki University Hospital and University of Helsinki, Helsinki, Finland

**Keywords:** B-lymphocyte, CD4, CD8, CD20, inflammation, saccular intracranial aneurysm, T-lymphocyte

## Abstract

Chronic inflammation in saccular intracranial aneurysm (sIA) walls is associated with wall rupture. Previous studies have shown an association between macrophages and CD3+ T-lymphocytes with sIA wall degeneration and rupture but the roles of T-lymphocyte subtypes and B-lymphocytes are poorly understood. Here, cyclic multiplexed immunofluorescent staining was used to investigate the presence of T-lymphocytes helper CD4+ and cytotoxic CD8+ T-lymphocytes and CD20+ B-lymphocytes in 16 unruptured and 20 ruptured sIA walls. The sIA walls contained both CD4+ (n = 25/35) and CD8+ (n = 21/35) subtypes. There was a greater density of CD4+ T-lymphocytes (median 7, range 0-642 cells/mm^2^) vs CD8+ T-lymphocytes (median 1, range 0-30 cells/mm^2^). The density of CD4+ T-lymphocytes was associated with wall rupture, the presence of an intraluminal thrombus and numbers of vascular and lymphatic neovessels. Additionally, the densities of CD20+ B- and CD4+ T-lymphocytes were associated with atherosclerosis- and inflammation-related changes, such as the accumulation of apolipoprotein B-100 and serum amyloid A, and densities of CD68+ and CD163+ macrophages. These findings suggest that CD4+ T-lymphocytes play a role in sIA rupture, and that CD4+ T- and CD20+ B-lymphocytes contribute to sIA wall inflammation, particularly in the presence of atherosclerotic changes.

## INTRODUCTION

Rupture of a saccular intracranial aneurysm (sIA) causes subarachnoid hemorrhage with high morbidity and mortality.^1^ Rupture-prone sIA walls are characterized by degenerative remodeling and chronic inflammation.^2–6^ Inflammatory cell infiltration is present within the walls of both unruptured and ruptured sIAs, indicating that inflammation is present before sIA rupture.^6–8^ Different inflammatory cell types, including macrophages (CD68+ and CD163+), neutrophils, T-lymphocytes (CD3+), memory T-lymphocytes (CD45ro+), B-lymphocytes (CD20+), natural killer cells, and mast cells have been detected in sIA walls.^2^^,^^3^^,^^8–11^ The numbers of CD3+ T-lymphocytes have been associated with rupture and degeneration of sIA walls,^2^^,^^11^ but there are only limited studies on the distribution and role of T-lymphocyte subtypes in the human sIA wall. Recently, a pediatric giant sIA was reported to harbor CD4+ and CD8+ T-lymphocytes,^12^ and compiled microarray data from 19 unruptured and 27 ruptured sIAs revealed that the ruptured IAs had higher proportions of CD4+ naïve T-lymphocytes than unruptured sIAs.^13^

All T-lymphocytes express CD3+ and can be divided into 2 major subtypes, namely CD8-expressing cytotoxic T-lymphocytes and CD4-expressing helper T-lymphocytes.^14^ CD8+ T-lymphocytes react to foreign antigens presented on major histocompatibility complex (MHC) I receptors by a wide range of normal tissue cells, after which they secrete cytokines inducing apoptosis of the antigen-presenting cell (APC).^14^ CD4+ T-lymphocytes detect antigens presented by MHC II receptors on APCs in draining lymph nodes. When exposed to secondary stimuli, CD4+ T-lymphocytes differentiate into further subtypes, of which Th1 and Th2 lymphocytes can stimulate B-lymphocytes to differentiate into either antibody-secreting plasma cells or memory cells.^15^ Th1 lymphocytes also help activate macrophages at sites of inflammation.^15^

Accumulating evidence points to a crucial role of Th1 and Th17 lymphocytes in the development and progression of atherosclerosis, which contribute to the chronic inflammation observed in atherosclerotic lesions.^16^^,^^17^ Similar changes as in atherosclerosis, such as accumulation of apolipoprotein B-100 and oxidatively and otherwise modified lipids, have been detected in degenerated and ruptured sIA walls.^4^^,^^18^

There is also evidence that Th2 and Th17 T-lymphocytes may enhance angiogenesis in ischemic injuries both in vivo and in vitro by directly stimulating endothelial sprouting of vascular neovessels.^19^ Vascular and lymphatic neovessels have been detected in sIA walls and it is hypothesized that leaky vascular neovessels may contribute to intramural microhemorrhages, as they have been shown to colocalize with these microhemorrhages and are associated with sIA wall degeneration.^3^^,^^20^ Moreover, vascular and lymphatic neovessels in human sIAs have been reported to be associated with markers of chronic inflammation,^20^^,^^21^ suggesting that inflammation plays a role in the neovascularization.

Abdominal aortic aneurysms (AAAs) and intracranial aneurysms (IAs) exhibit significant inflammatory cell infiltration, although the composition of the cells differs between AAAs and IAs. In AAAs, T-lymphocytes, rather than macrophages, are the major leukocyte subset in terms of cell number^22^; in sIAs, macrophages are more numerous than T-lymphocytes.^3^ Regarding T-lymphocyte subsets, CD4+ T-lymphocytes are more numerous than CD8+ T-lymphocytes in human AAAs.^22^ In transcriptome sequencing analysis of ruptured AAA tissue, the proportions of CD4+ memory T-lymphocytes, both resting and activated, and naïve B-lymphocytes are significantly higher when compared to other inflammatory cell types.^23^ In contrast, stable AAAs contain more memory B-lymphocytes and activated mast cells than other inflammatory cell types.^23^

This study used cyclic multiplexed immunofluorescence staining to analyze the presence of the major T-lymphocyte subtypes, CD4+ and CD8+, and CD20+ B-lymphocytes in a total of 36 ruptured and unruptured human sIA walls. The densities of these lymphocyte subsets were correlated with the presence or number of other inflammatory cells and markers, lipid accumulation markers, and markers of angiogenesis and lymphangiogenesis in the walls to investigate their potential roles in the pathogenesis of sIAs.

## METHODS

### sIA samples

A previously published sIA series of 16 unruptured and 20 ruptured sIA samples was studied.^3–5^^,^^20^^,^^24^^,^^25^ The sIA samples were acquired following surgical clipping at the Department of Neurosurgery, Helsinki University Hospital (HUH), Helsinki, Finland. After resection, the samples were rapidly frozen in liquid nitrogen and stored at −80°C. The frozen samples were embedded in Tissue-Tek (Sakura, Alphen aan den Rijn, the Netherlands) and were then cryosectioned at 4 μm for immunostaining. Clinical data were gathered from the patients’ medical records. sIA dimensions were derived from preoperative computed tomography angiography images. The HUH Ethics Committee approved this study.

### Basic characteristics of sIA samples

sIA samples were categorized as wall types A to D based on histology and features of degeneration as previously described by Ollikainen et al.^3^ and using the classification criteria by Frösen et al.^2^ Type A represents a wall with an intact endothelium and a well-organized layer of smooth muscle cells (SMCs); type B is characterized by a thickened wall with a disorganized SMCs and the absence of endothelium; type C displays a hypocellular wall with either myointimal hyperplasia or an organized thrombus; and type D corresponds to an extremely thin, hypocellular wall with an organized thrombus. Markers of inflammation, lipid accumulation, angiogenesis, and lymphangiogenesis in this sample series have been previously studied and analyzed,^3–5^^,^^24^ as defined in Table S1 and with some examples of these stainings in Figure S1. These previously published data were also included in the analyses with the new data. In these studies, positively stained areas were either calculated as percentage of the total wall area of the sIA wall section using ImageJ (NIH Software) or analyzed semiquantitatively dividing the samples in groups depending on extent of the staining. In the latter case, an increase in the stained marker was referred to as accumulation.

### Cyclic multiplexed immunofluorescence staining

Cyclic multiplexed immunofluorescence staining was used to perform multiple stainings for several antigens in one tissue section. The multi-step staining protocol is described in detail in the Supplemental Data File. The primary antibodies used are shown in Table S1.

Freshly frozen human tonsil tissue served as positive controls (Figure S2). An irrelevant mouse monoclonal antibody (IgG1; Serotec, UK) served as a substitute for the primary antibody in negative control stainings, which were also performed on the tonsil slides (Figure S2).

### Analysis

The sections were first scanned after CD3, CD4, CD8, and CD20 multiplexed immunofluorescent stainings and subsequently after the CD68 and CD163 multiplexed stainings with a Pannoramic 250 Flash III (3DHistec, Budapest, Hungary) to create images including each staining as a separate channel on the same section. The sIA sections were viewed and the wall areas were measured using a 3DHistec Slide Viewer (version 2.5).

The numbers of positively stained cells in the sIA samples were counted separately from the wall and the thrombus. For CD4 and CD8 stainings, only cells double-positive for both CD3 and CD4 or CD3 and CD8 were defined as positively stained for CD4 or CD8 T-lymphocytes, respectively. The densities of positively stained CD3+, CD4+, CD8+, CD20+, CD68+, and CD163+ cells in the sIA wall (presented as cells/mm^2^) were calculated by measuring the wall areas (range 0.26-4.95 mm^2^) excluding the thrombus and dividing the number of positively stained cells by the wall area. In sIA walls containing either or both CD4+ and CD8+ T-lymphocytes, it was determined whether the number of CD4+ or CD8+ T lymphocytes was higher, defined as the dominance of CD4+ or CD8+ T-lymphocytes in that wall. The cell densities and dominance of CD4+ or CD8+ T-lymphocytes in the sIA walls were compared with clinical and other histological parameters of the sIAs as previously described.^3–5^^,^^20^^,^^24^^,^^25^ The total number of analyzed samples was 35, as the histological section of a single sIA sample detached from the slide during the stainings.

### Statistics

Data analyses were performed using IBM SPSS Statistics Software, version 28. Fisher’s exact test was used for categorical variables. Kruskal-Wallis multiple comparison test, Mann-Whitney *U* test, and Spearman correlation test were used for continuous variables. Due to the large number of analyzed variables compared with the limited number of cases, the false discovery rate (FDR) procedure was used to mitigate false-positive *P* values. FDR-adjusted *P* values <.05 were considered statistically significant.

## RESULTS

### Distribution of inflammatory cells in sIA walls

**FIGURE 1.**
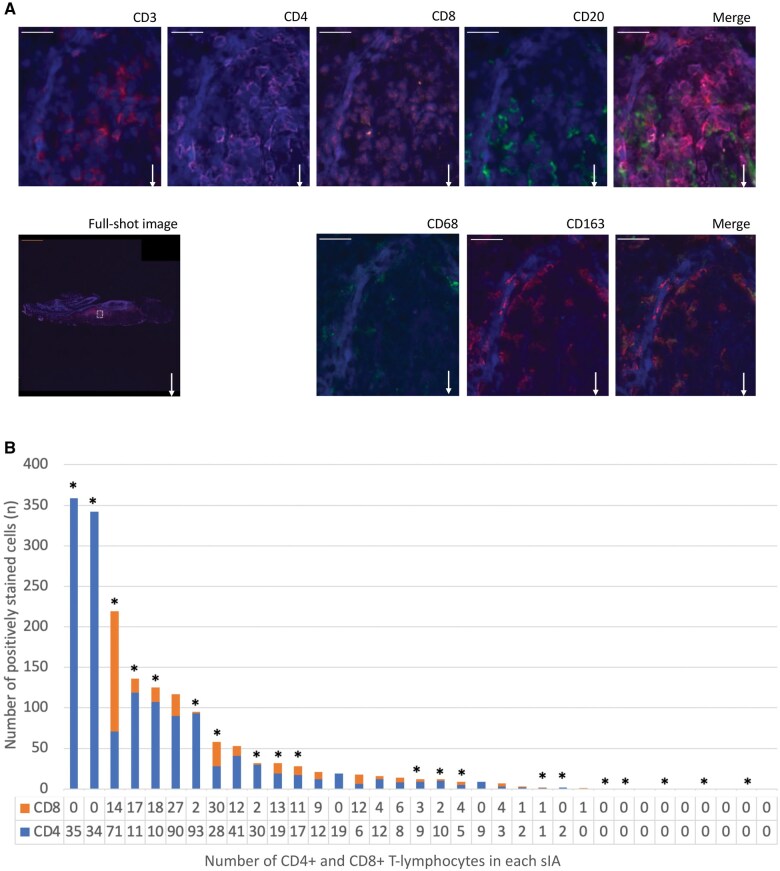
(A) Representative images of multiplexed immunofluorescence stainings for CD3 (red), CD4 (pink), CD8 (orange), CD20 (green), CD68 (green), and CD163 (red) in one ruptured saccular intracranial aneurysm (sIA) wall. Negative controls in tonsils are shown in Figure S2. The dashed square in the full-shot image of the sIA wall indicates the location of the close-up images. White arrows point down towards the lumen. Scale bars: 20 μm (white) and 500 μm (orange). (B) The absolute numbers of CD4+ (blue) and CD8+ (orange) T-lymphocytes in each of the 35 saccular intracranial aneurysm (sIA) samples. Each column represents an individual sample. Asterisk indicates a ruptured sIA sample.

In the 35 sIA walls examined, CD3+ (n = 28; 80%), CD4+ (n = 25; 71%), CD8+ (n = 21; 60%), CD20+ (n = 14; 40%), CD68+ (n = 30; 86%), and CD163+ (n = 30, 86%) cells were present (Figure 1A). All studied leukocyte types were scattered across the sIA walls; no preference for luminal or adventitial location was detected. The mean, median, and range for CD3+, CD4+, CD8+, CD20+, CD68+, and CD163+ cell densities in the sIA walls are presented in Table 1 and in Figure S3. In general, the CD20+ B-lymphocytes were detected in fewer samples and smaller quantities than the other studied leukocytes (Table 1). Consistent with our previous studies, CD163+ macrophages were the most abundant of the studied leukocytes (Table 1). Of the 7 aneurysms lacking detectable CD3^+^ cells, the majority (5 out of 7) were classified as wall type A, resembled normal arterial walls, and lacked other types of inflammatory cells. A box plot illustrating the distribution of the studied leukocytes across different sIA wall types is shown in Figure S3.

**Table 1.** Numbers of positively stained saccular intracranial aneurysm (sIA) samples and mean, median, and range of leukocyte densities in CD3+, CD4+, CD8+, CD20+, CD68+, and CD163+ stainings.

		CD3+ T-lymphocytes	CD4+ T-lymphocytes	CD8+ T-lymphocytes	CD20+ B-lymphocytes	CD68+ macrophages	CD163+ macrophages
Samples, n	Valid	35	35	35	35	35	35
	Missing	1	1	1	1	1	1
Positively stained sIA samples, n (%)		28 (80)	25 (71)	21 (60)	14 (40)	30 (86)	30 (86)
Leukocyte density, mean (cells/mm^2^)		75	45	4	8	82	88
Leukocyte density, median (cells/mm^2^)		24	7	1	0	27	46
Leukocyte density, range (cells/mm^2^)		0-794	0-642	0-30	0-129	0-750	0-565

Of the sIA walls, 25/35 (71%) contained either CD4+ or CD8+ lymphocytes or both. In 20/25 (80%) of the sIA walls containing CD4+ or CD8+ lymphocytes or both, the numbers of CD4+ T-lymphocytes were higher (median 12 cells, range 0-359 cells) than the numbers of CD8+ T-lymphocytes (median 4 cells, range 0-148 cells), indicating that helper T-cells were more abundant than the cytotoxic T-cells in most of the sIA walls (Figure 1B).

### Rupture, presence of thrombus, and vascular and lymphatic neovessels

**FIGURE 2.**
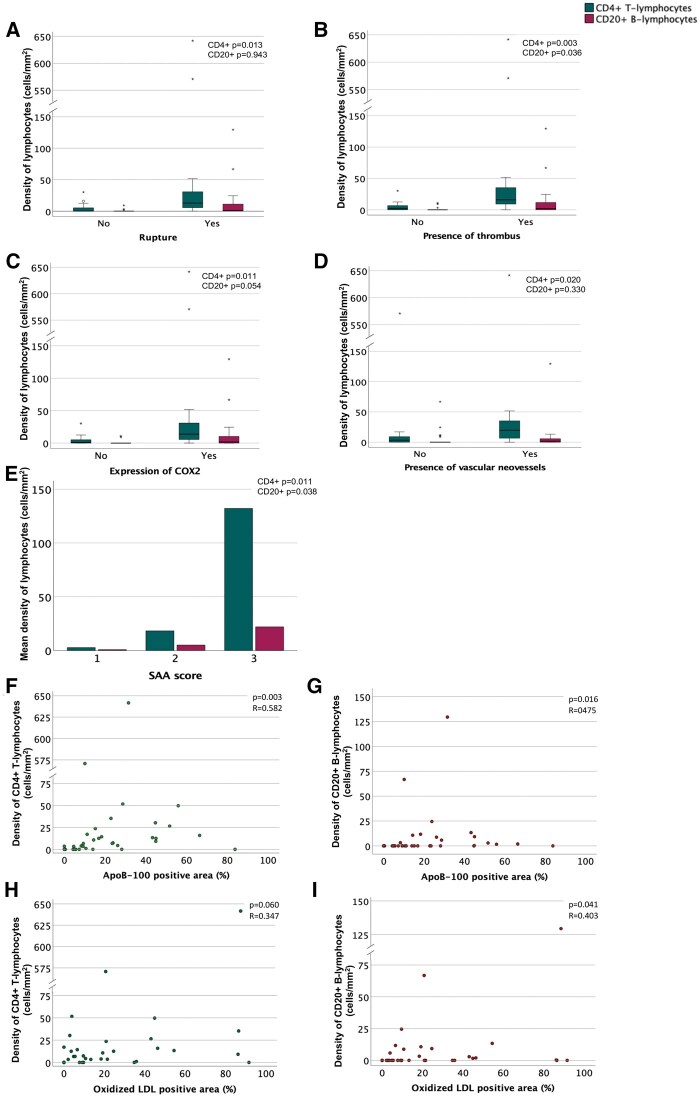
Distributions of the densities of CD4+ (green) and CD20+ (red) lymphocytes with the rupture status (A), presence of intraluminal thrombus (B), expression of cyclo-oxygenase 2 (COX-2) (C), presence of vascular neovessels (D), accumulation of serum amyloid A (SAA) (E), accumulation of apolipoprotein B-100 (apoB-100) (F and G), and oxidized low-density lipoprotein (LDL) (H and I) in the saccular intracranial aneurysm wall. Mann-Whitney *U*, Kruskal-Wallis, and Spearman tests were used in statistical analysis for nominal, categorical, and continuous variables, respectively.

Associations between densities of CD3+, CD4+, CD8+, CD20+, CD68+, and CD163+ cells with other sIA characteristics are shown in Table 2. A high density of CD4+ T-lymphocytes was associated with sIA wall rupture (Figure 2A). Furthermore, the densities of CD4+ T-lymphocytes and CD20+ B-lymphocytes were positively associated with the presence of thrombus (Figure 2B). However, analyzing unruptured and ruptured sIAs separately, no significant associations were found between the presence of thrombus and CD4+ and CD20+ lymphocyte densities, although visual assessment of the data indicated that unruptured sIAs without thrombus exhibited lower densities of CD4+ and CD20+ lymphocytes, whereas ruptured sIAs with thrombus demonstrated higher densities of these lymphocyte populations. Also, the densities of CD8+ T-lymphocytes and the dominance of CD4+ T-lymphocytes over CD8+ T-lymphocytes were not associated with the presence of thrombus.

**Table 2.** Associations of inflammatory cell densities with other histological characteristics of the saccular intracranial aneurysm (sIA) walls.

Variable^a^	Density of positive cells in the sIA wall (cells/mm^2^)						
	CD3+ T-lymphocytes	CD4+ T-lymphocytes (ie, CD4+ and CD3+ double-positive cells)	CD8+ T-lymphocytes (ie, CD8+ and CD3+ double-positive cells)	CD20+ B-lymphocytes	CD68+ macrophages	CD163+ macrophages	**CD4+/CD8+ lymphocyte dominance** ^b^
**Characteristics of the sIA**							
Rupture	**0.002** ^§^	**0.013** ^§^	NS^§^	NS^§^	NS^§^	NS^§^	NS^†^
Wall type	NS^‡^	NS^‡^	NS^‡^	NS^‡^	NS^‡^	NS^‡^	NS^†^
Presence of thrombus	**<0.001** ^§^	**0.003** ^§^	NS^§^	**0.038** ^§^	**0.007** ^§^	NS^§^	NS^†^
Presence of vascular (CD34+ or CD31+) neovessels	**0.016** ^§^	**0.020** ^§^	NS^§^	NS^§^	**0.013** ^§^	**0.005** ^§^	NS^†^
Density of vascular (CD34+) neovessels/sIA wall area (n/mm^2^)	**0.038** ^#^	**0.013** ^#^	NS^#^	NS^#^	**0.013** ^#^	**0.011** ^#^	NS^†^
Number of (Prox1+, LYVE-1+, podoplanin+, or VEGFR-3+) lymphatic neovessels	NS^#^	**0.013** ^#^	NS^#^	NS^#^	**0.007** ^#^	**0.011** ^#^	NS^†^
Extent of smooth muscle cells	NS^‡^	NS^‡^	NS^‡^	NS^‡^	**0.016** ^c^ ^‡^	NS^‡^	NS^†^
**Density of inflammatory cells in the sIA wall (cells/mm^2^)**							
Mast cells	NS^#^	NS^#^	NS^#^	NS^#^	NS^#^	**0.008** ^#^	NS^§^
CD3+ T-lymphocytes		**<0.001** ^#^	NS^#^	**<0.001** ^#^	**0.003** ^#^	**0.003** ^#^	**0.005** ^§^
CD4+ T-lymphocytes	**<0.001** ^#^		**0.020** ^#^	**0.003** ^#^	**0.011** ^#^	**0.020** ^#^	**<0.001** ^§^
CD8+ T-lymphocytes	NS^#^	**0.020** ^#^		NS^#^	NS^#^	NS^#^	**0.005** ^§^
CD20+ B-lymphocytes	**<0.001** ^#^	**0.003** ^#^	NS		**0.016** ^#^	**0.020** ^#^	**0.039** ^§^
CD68+ macrophages	**0.003** ^#^	**0.011** ^#^	NS^#^	**0.016** ^#^		**<0.001** ^#^	NS^§^
CD163+ macrophages	**0.003** ^#^	**0.020** ^#^	NS^#^	**0.020** ^#^	**<0.001** ^#^		NS^§^
***Inflammatory markers in the sIA wall***							
SAA accumulation	**0.008** ^‡^	**0.011** ^‡^	**0.043** ^‡^	**0.038** ^‡^	**<0.001** ^‡^	**0.008** ^‡^	NS^†^
COX-2 expression	**0.003** ^§^	**0.011** ^§^	NS^§^	NS^§^	**0.006** ^§^	NS^§^	**0.045** ^†^ (CD4)
MPO accumulation	NS^‡^	NS^‡^	NS^‡^	NS^‡^	**0.030** ^‡^	NS^‡^	NS^†^
MMP-9 accumulation	**0.037** ^‡^	**0.067** ^‡^	NS^‡^	NS^‡^	**0.011** ^‡^	**0.034** ^‡^	NS^†^
MMP-9 expression	**0.020** ^§^	**0.022** ^§^	NS^§^	NS^§^	**0.013** ^§^	**0.030** ^§^	NS^†^
Glycophorin A accumulation	**0.035** ^‡^	**0.049** ^‡^	NS^‡^	NS^‡^	**0.020** ^‡^	NS^‡^	NS^†^
HO-1 accumulation	**0.028** ^‡^	NS^‡^	NS^‡^	**0.049** ^‡^	NS^‡^	**0.039** ^‡^	NS^†^
HLA-DR accumulation	NS^‡^	NS^‡^	NS^‡^	NS^‡^	**0.022** ^‡^	**0.038** ^‡^	NS^†^
***Area (%) of lipid accumulation markers in the sIA wall***							
ORO	NS^#^	NS^#^	NS^#^	NS^#^	NS^#^	NS^#^	NS^§^
ApoA-1	NS^#^	NS^#^	NS^#^	NS^#^	**0.022** ^#^	NS^#^	NS^§^
ApoB-100	**<0.001** ^#^	**0.003** ^#^	NS^#^	**0.016** ^#^	**0.008** ^#^	**0.013** ^#^	**0.037** ^§^ (CD4)
Oxidized LDL	NS^#^	NS^#^	NS^#^	**0.041** ^#^	**0.013** ^#^	NS^#^	NS^§^
Adipophilin	**0.008** ^#^	**0.035** ^#^	NS^#^	NS^#^	**0.011** ^#^	**0.020** ^#^	NS^§^

FDR-corrected *P* values < .05 were considered statistically significant.

“Presence” and “expression” refer to binary variables, “extent” and “accumulation” refer to semiquantitative analysis of ordinal variables, and area,” “number,” and “density” refer to continuous variables, as defined in previous works.^3–5^^,^^24^

CD, cluster of differentiation; SAA, serum amyloid A; MPO, myeloperoxidase; COX-2, cyclo-oxygenase 2; MMP-9, matrix metalloproteinase 9; ORO, Oil-Red O, ie neutral lipids; ApoA-1, apolipoprotein A-1; ApoB-100, apolipoprotein B-100; LDL, low-density lipoprotein; LYVE-1, lymphatic vessel endothelial hyaluronic acid receptor-1; Prox1, prospero-related homeobox 1; HO-1, heme oxygenase 1; HLA-DR, human leukocyte antigen-DR isotype.

b In samples double-positive for both CD4+ and CD8+ T-lymphocytes.

c The association between the density of CD68+ macrophages and extent of smooth muscle cells were negative. All other associations were positive.

† Fisher exact test.

‡ Kruskal-Wallis test.

§ Mann-Whitney *U* test.

# Spearman test.

There was a positive correlation between numbers of vascular and lymphatic neovessels in the sIA walls and densities of CD4+ T-lymphocytes (Figure 2D). In addition, when analyzing the unruptured and ruptured sIAs separately, CD4+ T-lymphocytes and CD20+ B-lymphocytes were significantly associated with the presence of both vascular (*P* = .035 and *P* = .044, respectively) and lymphatic neovessels (*P* = .009 and *P* = .026, respectively) in unruptured sIAs. In ruptured sIAs, visual assessment suggested higher lymphocyte densities in samples with neovessels but no statistically significant associations were observed; this is likely due to one outlier sample lacking neovessels yet showing high lymphocyte levels, which strongly influenced the analysis in this limited sample size.

### Other inflammatory cells and markers

The densities of CD4+ T-lymphocytes and CD20+ B-lymphocytes positively correlated with the densities of CD68+ or 163+ macrophages in the sIA walls. Moreover, the densities of CD4+ T-lymphocytes, CD8+ T-lymphocytes, and CD20+ B-lymphocytes were positively associated with the accumulation of serum amyloid A (SAA) in the sIA wall (Figure 2E). The density and predominance of CD4+ T-lymphocytes also showed a positive association with expression of cyclo-oxygenase-2 (COX-2; Figure 2C). Additionally, the density of CD4+ T-lymphocytes was positively associated with both the expression and accumulation of matrix metalloproteinase-9 (MMP-9) (Table 2).

### Accumulation of lipids and red blood cells

The densities of CD4+ T-lymphocytes and CD20+ B-lymphocytes and the predominance of CD4+ T-lymphocytes were positively associated with extensive positive staining for apolipoprotein B-100 (apoB-100) in the sIA walls (Figure 2F and G). In addition, the density of CD20+ B-lymphocytes was positively correlated with the extent of oxidized lipids (hydroxynonenal-positive area; Figure 2H and I). Furthermore, the densities of CD4+ T-lymphocytes positively correlated with adipophilin, a marker of intracellular lipid accumulation.

The density of CD4+ T-lymphocytes was positively associated with the extent of glycophorin A-positive staining (a marker for red blood cell debris) in the sIA walls. In addition, the density of CD20+ B-lymphocytes exhibited a positive association with heme oxygenase-1 (HO-1; an enzyme that breaks down heme) in the sIA walls.

## DISCUSSION

This study revealed the presence and distribution of CD4+ helper- and CD8+ cytotoxic T-lymphocyte subtypes in a series of 35 adult human sIA walls. CD4+ T-lymphocytes were identified in 71% and CD8+ T-lymphocytes in 60% of the sIA walls. Notably, there was a preponderance of CD4+ T-lymphocytes in most cases where both CD4+ and CD8+ T-lymphocytes coexisted, suggesting that helper T-lymphocytes may have a more significant role in the inflammation of sIA walls than cytotoxic T-lymphocytes.

**FIGURE 3.**
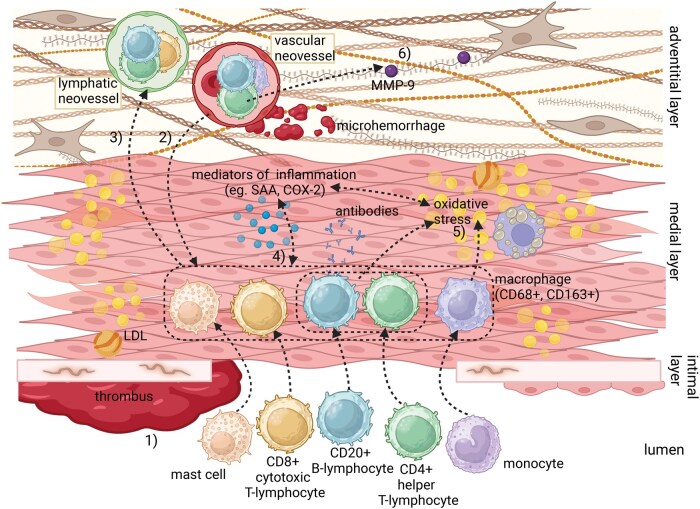
Possible interactions of T- and B-lymphocytes within the saccular intracranial aneurysm (sIA) wall. During the development of a sIA, an intraluminal thrombus starts to form at the site of disrupted endothelium and lipids, such as LDL containing apoprotein B-100 (apoB-100), begin to accumulate in the sIA wall, which may attract inflammatory cells to invade the wall.^4^^,^^6^^,^^7^ CD4+ helper T-lymphocytes, CD8+ cytotoxic T-lymphocytes, CD20+ B-lymphocytes, monocytes, mast cells, and macrophages (CD68+, CD163+) may infiltrate the sIA wall either (1) via the lumen or (2) through vascular neovessels. Leaky vascular neovessels may contribute to microhemorrhages, which can further enhance the inflammatory response. (3) Lymphatic neovessels could facilitate the transport of B- and T-lymphocytes and proinflammatory mediators to lymph nodes, where further immune activation occurs.^20^ (4) Within the sIA wall, T- and B-lymphocytes, macrophages, mast cells, and inflammatory mediators such as cyclo-oxygenase 2 (COX-2) and serum amyloid A (SAA), interact with each other further enhancing inflammation and immune cell recruitment.^3^^,^^5^^,^^6^^,^^25^ (5) The accumulated lipids in the sIA wall are oxidized and modified, and interactions between CD4+ T-lymphocytes, B-lymphocytes, and other mediators of inflammation may enhance this process.^4^^,^^18^ The accumulation of oxidized lipids within the sIA wall might also stimulate B-lymphocyte-mediated antibody production against these modified lipids.^18^ However, the impact of T- and B-lymphocytes on atherosclerotic changes within sIA wall might depend on their specific subsets, which may exert either pro-atherogenic or athero-protective effects.^16^^,^^17^ (6) MMP-9 (matrix metalloproteinase-9) released from, eg CD4+ T-lymphocytes, leads to extracellular matrix degradation, weakening the aneurysmal wall and predisposing it to rupture.^27^ The figure was created with BioRender.com.

The function of T-lymphocytes in intracranial aneurysm formation has been studied in an experimental rat model, in which neither the deficiency in T-lymphocytes nor pharmacological inhibition of T-lymphocyte function had any impact on sIA progression, suggesting that T-lymphocyte function would not be required for sIA formation at least in this animal model.^26^ In the current human study, the high densities of CD4+ T-lymphocytes were associated with sIA rupture, which suggests involvement of these lymphocytes in the processes that lead to a rupture-prone sIA. Although no associations between sIA wall degeneration and lymphocytes were detected in the current study, the density of CD4+ T-lymphocytes was associated positively with multiple variables known to contribute to sIA wall remodeling, such as accumulation of apoB-100 and oxidatively or otherwise modified LDL, presence of intraluminal thrombus, and expression of MMP-9, COX-2, and SAA.^2^^,^^4^^,^^25^ These results support the notion that CD4+ helper T-lymphocytes contribute to the pathogenesis of sIA, particularly in inflammatory and atherosclerotic alterations such as accumulation of lipids and leukocytes in the sIA wall. Figure 3 summarizes the possible effects of CD4+ T-lymphocytes and CD20+ B-lymphocytes in the sIA wall.

Atherosclerotic changes of human sIA walls are abundant and are associated with both aneurysm wall degeneration and rupture.^18^ Accumulation of oxidized lipids in the vasculature is known to initiate an immune response, which can result in the formation of antibodies against these lipids.^28^ Plasma IgG antibody levels reactive to oxidized LDL are higher in patients with unruptured sIAs than in patients with aneurysmal SAH,^18^ suggesting that antibodies produced by B-lymphocytes against oxidized lipids may have a protective effect against sIA rupture. However, studies in atherosclerotic mouse models revealed that the effects of T- and B-lymphocytes on the progression of atherosclerosis depend on their subsets, which may be either atherogenic or protective against atherosclerosis.^16^^,^^17^ In addition, crosstalk between CD4+ T-lymphocytes and B-lymphocytes via MHC II and CD40 molecules increases development of atherosclerosis.^29^ Thus, although both CD4+ helper T-lymphocytes and CD20+ B-lymphocytes may have a role in pathologic lipid accumulation in sIA walls, further studies are needed to identify the individual roles of their subsets in this process.

Vascular and lymphatic neovessels are present especially in sIA walls with atherosclerotic changes.^3^^,^^20^ The results of the present study suggest potential interactions between CD4+ helper T-lymphocytes and lymphangiogenesis or angiogenesis in the sIA wall. In addition, the presence of CD4^+^ and CD20^+^ lymphocytes increased in parallel with both aneurysm rupture and the presence of vascular and lymphatic neovessels: unruptured sIAs without neovessels exhibited virtually no lymphocytes, whereas those with neovessels showed low densities. In ruptured sIAs, lymphocyte densities were modest in the absence of neovessels but markedly elevated when neovessels were present. As lymphatic vessels transport leukocytes to lymph nodes, in addition to other mediators of inflammation, these vessels could transport CD4+ helper T-lymphocytes and proinflammatory substances produced by T-lymphocytes from the sIA wall to the lymph nodes, where B-lymphocytes and cytotoxic T-lymphocytes may be activated and transported back to the sIA walls via the luminal circulation or via the vascular neovessels.

Angiogenesis requires endothelial cells to penetrate the extracellular matrix, a process that requires proteolytic degradation of matrix molecules, eg via the action of MMPs.^30^ In this respect, it is relevant that T-lymphocytes can contribute to angiogenesis by inducing expression of MMPs in endothelial cells.^31^ In this study, CD4+ T-lymphocytes were positively associated with the expression and accumulation of MMP-9, suggesting their potential contribution to extracellular matrix degradation and ensuing destabilization of the sIA wall. Nevertheless, it is essential to consider that T-lymphocytes also produce MMP-9, which may contribute to the observed positive association between MMP-9 and CD4+ T-lymphocytes.

Although the densities of both CD4+ T-lymphocytes and CD20+ B-lymphocytes had mutually similar associations with inflammatory and atherosclerotic changes in the sIA walls, the density of CD8+ T-lymphocytes was associated exclusively with accumulation of SAA. This may reflect differences in their roles or significance in sIA formation. Considering that CD4+ helper T-lymphocytes primarily activate other lymphocytes, such as B-lymphocytes, and that cytotoxic CD8+ T-lymphocytes directly eliminate infected and malignant cells, the differences observed in their associations may stem from their separate functions within the immune system. Likewise, the similar associations observed between B-lymphocytes and CD4+ T-lymphocytes in sIA walls may be explained by the required B-lymphocyte activation through CD4+ T-lymphocytes.

### Limitations

In this study, each histologic section exhibited pathological changes only in one section of the aneurysm wall and thus may not reflect the changes in the entire wall. This may explain why some of our samples were negative for certain inflammatory cell types, in contrast to the microarray analysis by Li et al. that did not reveal similarly negative samples.^13^ Furthermore, this study cannot determine the extent to which these cells contribute to the degenerative remodeling of the aneurysm wall; therefore, further research is needed to clarify the precise mechanisms by which T-lymphocytes influence the sIA wall. However, this series allowed for a robust analysis of the associations of different inflammatory cell types in human sIA walls and their relationships in cyclic multiplexed immunostainings and permitted simultaneous histological study.

## CONCLUSION

This is the first demonstration of the presence of CD4+ and CD8+ T-lymphocyte subtypes and their histological correlations with other inflammatory cells and atherosclerotic changes in human sIA walls. Among these subtypes, CD4+ T-lymphocytes were more abundant than CD8+ T-lymphocytes and were associated with sIA wall rupture and various characteristic pathological features of sIA walls. This suggests a more significant role for helper T-lymphocytes than cytotoxic T-lymphocytes in the pathogenesis of sIAs. The densities of CD4+ T-lymphocytes and CD20+ B-lymphocytes were positively associated with increased lipid accumulation and inflammatory markers in the sIA walls, suggesting that they may contribute to the chronic inflammation and atherosclerotic changes present in the walls. However, further research is warranted to characterize the precise functions of immune cell subtypes and their individual roles in the progression and ultimate rupture of sIAs.

## Supplementary Material

nlaf060_Supplementary_Data
