# Supplementary material for: CD4+ T-lymphocytes in human saccular intracranial aneurysm walls are associated with aneurysm rupture
Source: J Neuropathol Exp Neurol. 2025 Jun 11;84(10):870–8. doi: 10.1093/jnen/nlaf060 (PMC12456882; doi:10.1093/jnen/nlaf060)
Supplement: nlaf060_Supplementary_Data [file nlaf060_supplementary_data.zip › Supplementary Data/Figure S1.pdf]

Supplemental Figure 1

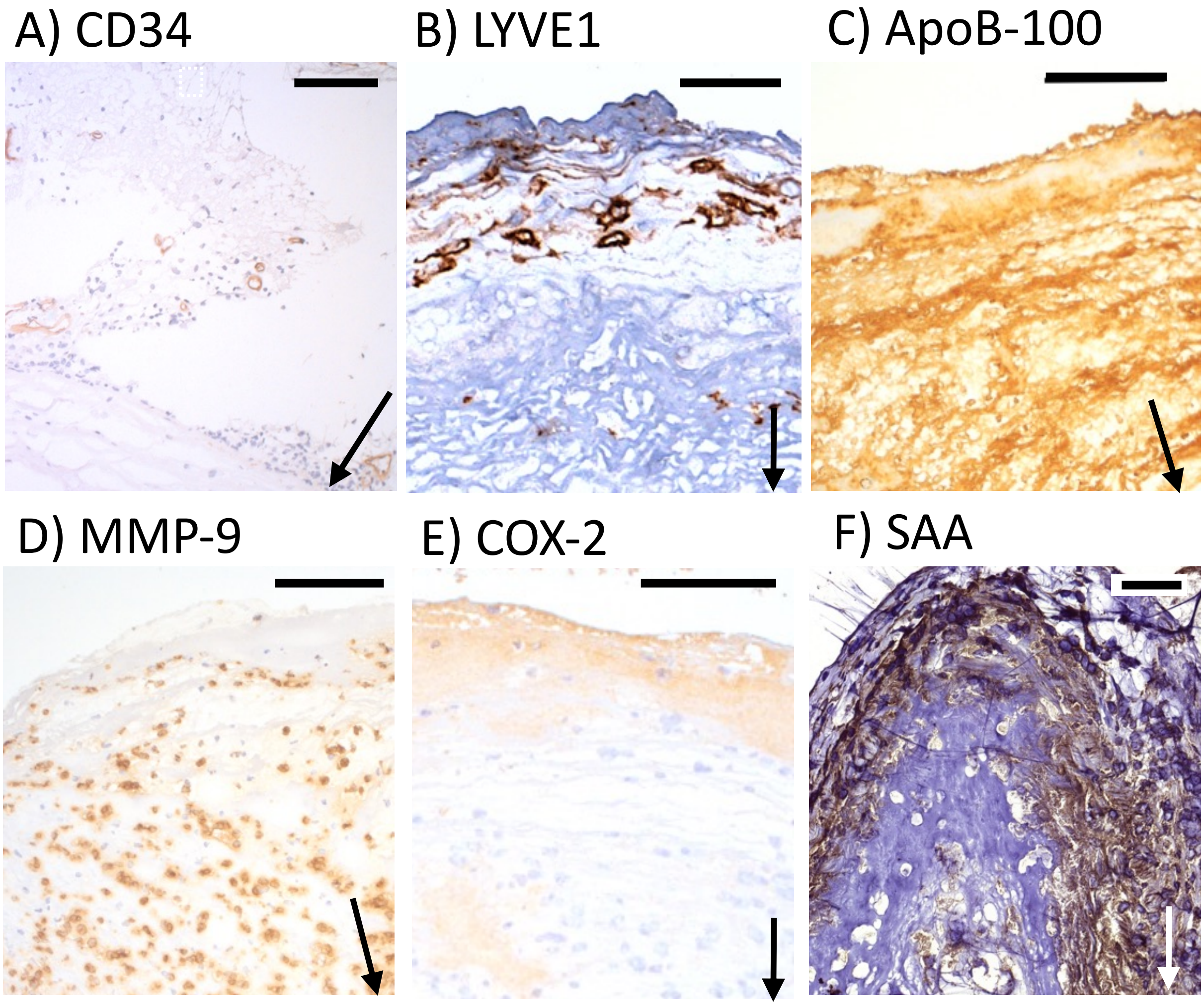

Representative images of immunohistochemical stainings for CD34+ vascular neovessels (A), LYVE-1+ lymphatic neovessels (B), apolipoprotein B-100 (ApoB-100; C), matrix-metalloproteinase-9 (MMP-9; D), cyclo-oxygenase 2 (COX-2; E), and serum amyloid A (SAA; F) in saccular intracranial aneurysm walls. Arrows point down towards the lumen. Positive staining is brown. Hematoxylin background staining. Scale bars: (A-B, F) 50 µm and (C-D) 100 µm.
