# Supplementary material for: CD4+ T-lymphocytes in human saccular intracranial aneurysm walls are associated with aneurysm rupture
Source: J Neuropathol Exp Neurol. 2025 Jun 11;84(10):870–8. doi: 10.1093/jnen/nlaf060 (PMC12456882; doi:10.1093/jnen/nlaf060)
Supplement: nlaf060_Supplementary_Data [file nlaf060_supplementary_data.zip › Supplementary Data/Figure S3.pdf]

Supplemental Figure 3

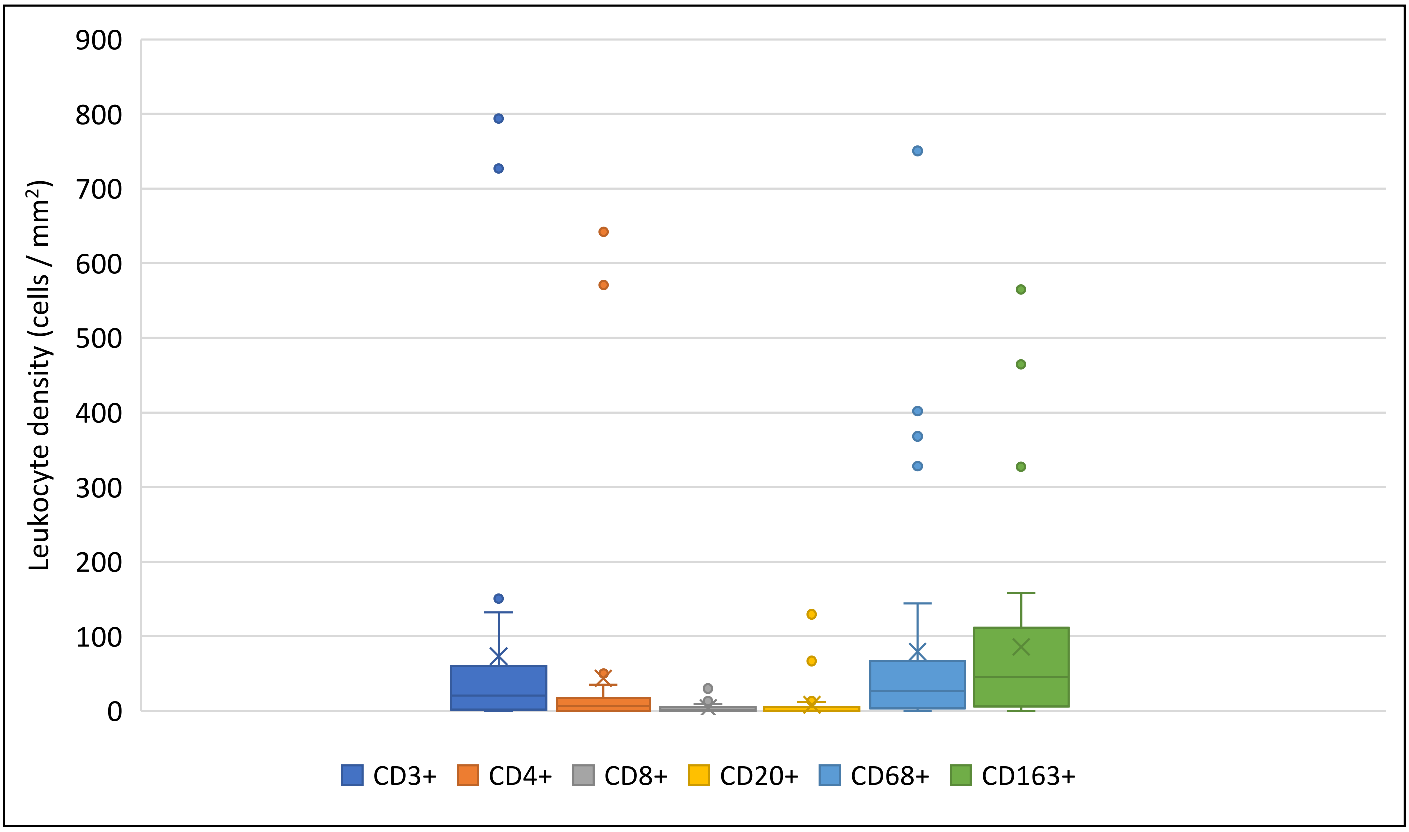

Leukocyte densities in CD3+, CD4+, CD8+, CD20+, CD68+, and CD163+ stainings in 35 saccular intracranial aneurysm walls. The horizontal line, which splits the box in two, denotes the median. The X on the box denotes the mean. The dots denote the outliers.

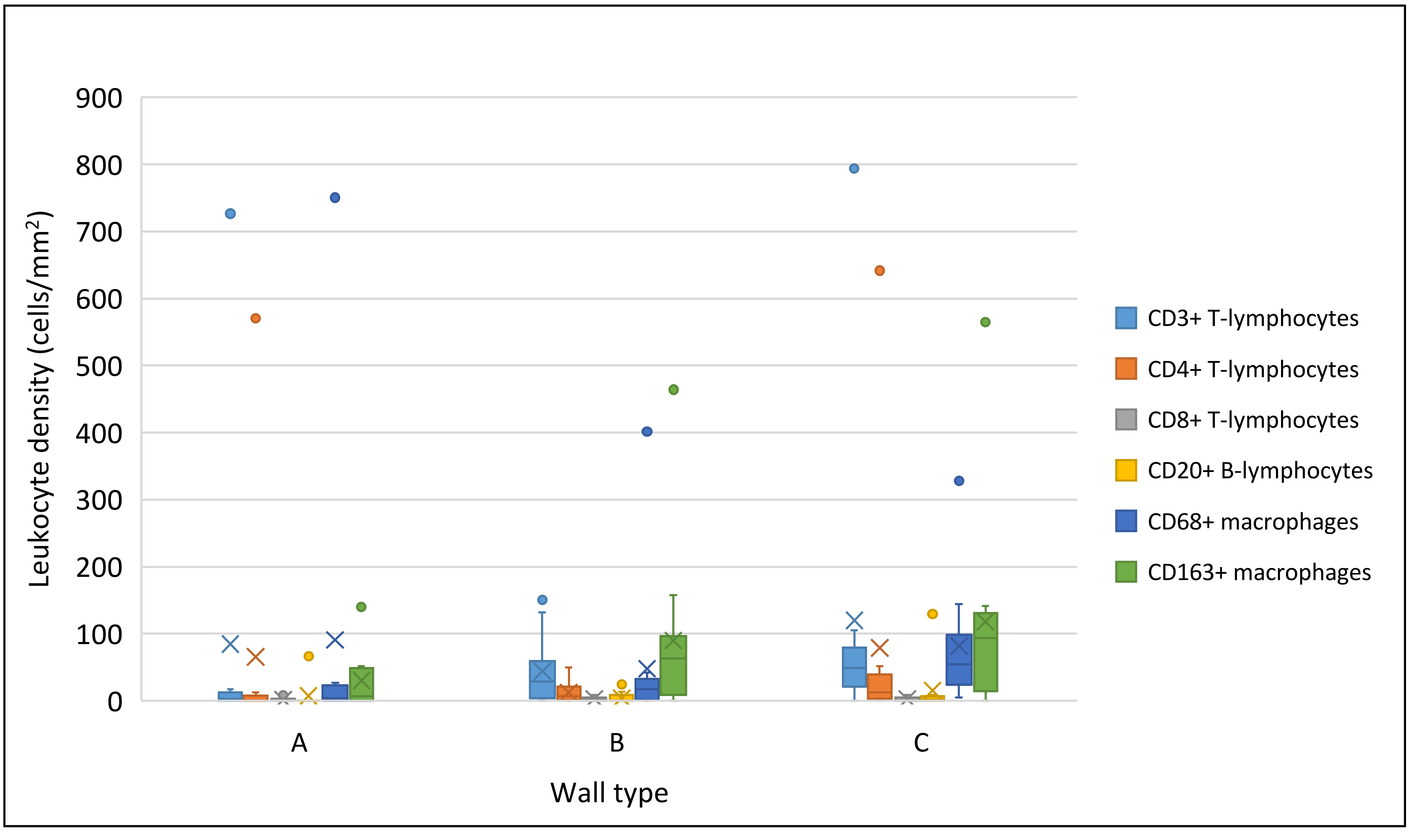

The distribution of the studied leukocyte densities in CD3+, CD4+, CD8+, CD20+, CD68+, and CD163+ stainings across different wall types in 35 saccular intracranial aneurysm walls. The horizontal line, which splits the box in two, denotes the median. The X on the box denotes the mean. The dots denote the outliers.
