# Supplementary material for: CD4+ T-lymphocytes in human saccular intracranial aneurysm walls are associated with aneurysm rupture
Source: J Neuropathol Exp Neurol. 2025 Jun 11;84(10):870–8. doi: 10.1093/jnen/nlaf060 (PMC12456882; doi:10.1093/jnen/nlaf060)
Supplement: nlaf060_Supplementary_Data [file nlaf060_supplementary_data.zip › Supplementary Data/Supplemental Data File.docx]

**SUPPLEMENTAL DATA:** Multiplexed immunofluorescent staining protocol.

The histological sections were fixed with 4 % paraformaldehyde (PFA) for 5 min at room temperature (RT), washed with phosphate-buffered saline (PBS) and Tris-buffered saline (TBS) for 3 min, and sequentially incubated with the peroxidase block (0.9 % peroxidase [Thermo Fisher Scientific, Carlsbad, CA, USA] in TBS) for 15 min and 10 % normal goat serum (NGS; Gibco, Auckland, New Zealand) for 15 min at RT. The primary mouse anti-human CD20 antibody and rabbit anti-human CD3 antibody (Supplementary file 1) were diluted to the buffer solution (10 % NGS in TBS with 0.05 % Tween) and incubated on the sections for 60-120 min at RT. The secondary detection for the mouse anti-human CD20 antibody was performed with Bright Vision horseradish peroxidase (HRP) reagent (WellMed B.W., Arnhem, the Netherlands) diluted 1:5 for 30 min in RT. TSA reagent (Tyramide fluorochrome 488 [Thermo Fisher Scientific, Eugene, OR, USA] 1:200 and peroxidase 1:20 000 in TBS with Tween), which binds to HRP, was applied for 15 min in RT. The sections were incubated again with the peroxidase block and after that secondary detection of rabbit anti-human CD3 antibody was performed with HRP and TSA reagent (with Tyramide fluorochrome 555 [Thermo Fisher Scientific, Eugene, OR, USA]). The sections were then incubated at 4^o^ C overnight.

The next day, the sections were heated in 10 mM Tris-1 mM EDTA pH 9 at 99 C for 20 min to denaturize the previous antibodies. In the second round, the sections were incubated in the 10 % NGS and then the primary rabbit anti-human CD4 and mouse anti-human CD8 antibodies (Supplemental file 1) were incubated on the sections for 60-120 min at RT. Secondary detection of these antibodies was performed using Alexa-647 goat anti-rabbit and Alexa-750 goat anti-mouse antibodies (Thermo Fisher Scientific, Eugene, OR, USA), respectively, diluted 1:300 in TBS with Tween and DAPI for nucleus detection (Roche, Mannheim, Germany), and incubating the sections 30 min in RT. The sections were mounted with ProlongGold mounting medium (Thermo Fisher Scientific, Eugene, OR, USA) and scanned.

The coverslips were soaked off in TBS and the sections were bleached by soaking them in a solution of 30 % peroxidase and 1 M sodium hydroxide in TBS for 30-60 min in RT and heated. Sections were blocked using NGS. The primary mouse anti-human CD68 and rabbit anti-human CD163 antibodies (Additional file 1: Table S1) were incubated on the sections for 60-120 min at RT. Secondary detection of these antibodies was performed using Alexa-647 goat anti-mouse and Alexa-750 goat anti-rabbit antibodies (Thermo Fisher Scientific, Eugene, OR, USA), respectively, diluted 1:300 in TBS with Tween and DAPI for nucleus detection, and incubating the sections 30-45 min in RT. The sections were mounted with ProlongGold mounting medium.
