# Supplementary material for: CD4+ T-lymphocytes in human saccular intracranial aneurysm walls are associated with aneurysm rupture
Source: J Neuropathol Exp Neurol. 2025 Jun 11;84(10):870–8. doi: 10.1093/jnen/nlaf060 (PMC12456882; doi:10.1093/jnen/nlaf060)
Supplement: nlaf060_Supplementary_Data [file nlaf060_supplementary_data.zip › Supplementary Data/Table S1.docx]

**SUPPLEMENTAL TABLE 1:** Primary antibodies used in the immunostainings.

| **Antigen*** | **Function** | **Antibody clone** | **IgG subclass** | **Manufacturer **** | **Concentration (mg/l) or final dilution** |
| --- | --- | --- | --- | --- | --- |
| **CD3** (I) | T-lymphocyte co-receptor, which is involved in activating both cytotoxic and helper T-lymphocytes. | SP7 | Rabbit  monoclonal  IgG1 | Novus  Biologicals | IF: 1:50 |
| **CD4** (I) | T-lymphocyte co-receptor, which is involved in activating helper T-lymphocytes. | EPR6855 | Rabbit monoclonal IgG1 | Abcam | IF: 1:200 |
| **CD8** (I) | T-lymphocyte co-receptor, which is involved in aiding cytotoxic T-lymphocyte interactions with antigens. | C8/144B | Mouse monoclonal  IgG1 | Dako | IF: 1:200 |
| **CD20** (I) | B-lymphocyte co-receptor, which enables B-lymphocyte immuno -response against antigens. | L26 | Mouse monoclonal  IgG1 | Thermo Fisher Scientific | IF: 1:500 |
| **CD68** (I) | Transmembrane glycoprotein highly expressed by cells in the monocyte lineage. | KP1 | Mouse  monoclonal  IgG1 | Cell Marque | IF: 1:100 |
| **CD163**(I) | A scavenger receptor for haptoglobin-hemoglobin complexes, which are expressed by monocyte/macrophage-line. | EPR14643 | Rabbit  monoclonal  IgG1 | Abcam | IF: 1:200 |
| **αSMA** (II) | An actin isoform, which is abundant in vascular smooth muscle cells and contributes to cell-generated mechanic tension. | 1A4 | Mouse monoclonal IgG2 | Dako | IHC: 1:1000 |
| **CD31** (II) | Cell-surface glycoprotein widely used as a marker for endothelial differentiation. Also known as PECAM-1. | JC70A | Mouse monoclonal IgG1 | Dako | IHC: 200 |
| **CD34** (II) | A transmembrane glycoprotein expressed on e.g. endothelial cells and used as a marker for vascular neovessels. | QBEnd/10 | Mouse monoclonal IgG1 | Novocastra | IHC: 12 |
| **Mast cell tryptase** (II) | Protein secreted and stored by mast cells. | AA1 | Mouse monoclonal IgG1 | Dako | IHC: 1:500 |
| **Mast cell chymase** (II) | Protein secreted and stored by mast cells. | CC1 | Mouse monoclonal IgG1 | Serotec | IHC: 1:500 |
| **SAA** (III) | A family of apolipoproteins, which are elevated in an acute-phase response. | MC1 | Mouse monoclonal IgG1 | Dako | IHC: 1:100 |
| **COX-2** (III) | An enzyme responsible for the formation of prostanoids, which are released in high amounts locally at the site of inflammation. | CX-294 | Mouse monoclonal IgG1 | Dako | IHC: 1:100 |
| **MPO** (IV) | A lysosomal enzyme expressed mainly in neutrophils and to a lesser degree in monocytes. | A0398 | Rabbit polyclonal | Dako | IHC: 1.8 |
| **MMP-3** (IV) | A protein involved in the breakdown of extracellular matrix. | - | Rabbit polyclonal | Abcam | IHC: 10 |
| **MMP-9** (IV) | A protein involved in the breakdown of extracellular matrix. | 4H3 | Mouse monoclonal IgG1 | Novus Biologicals | IHC: 0.2 |
| **Apolipoprotein A-I**(V) | A major protein component of the high-density lipoprotein complex, which enables the reverse cholesterol transport. | 1C5 | Mouse monoclonal IgG1 | Monosan | IHC: 0.01 |
| **Apolipoprotein B-100** (V) | A major protein component of the intermediate-, low- and very low-density lipoprotein complexes. | MB-47 | Mouse monoclonal IgG2a | ^1^ | IHC: 1:10 000 |
| **Oxidized LDL (hydroxynonenal**)(V) | Oxidization of low-density lipoproteins plays a key role in the development of the early atherosclerotic lesions. | HNE | Guineapig polyclonal | ^2^ | IHC: 1:300 |
| **Adipophilin**(V) | A protein on the surface of intracellular lipid droplets and used as a marker of intracellular lipid accumulation. | AP125 | Mouse monoclonal IgG1 | R&D Systems | IHC: 1:10 |
| **LYVE-1** (VI) | Cell surface receptor for hyaluronan. Enhances dendritic cell entry into the lymphatic capillaries. | - | Goat polyclonal | R&D Systems | IHC: 2 |
| **Podoplanin** (VI) | Cell surface receptor, which binds to C-type lectin-like receptor 2 on platelets. Plays a role in lymphatic separation from the blood vasculature. | D2-40 | Mouse monoclonal IgG1 | Dako | IHC: 0.285 |
| **Prox1** (VI) | Transcription factor, which indicates beginning of the differentiation of lymphatic structures. | - | Goat polyclonal | R&D Systems | IHC: 2 |

* CD (cluster of differentiation), αSMA (alpha-smooth muscle actin), SAA (serum amyloid A), COX-2 (cyclo-

oxygenase-2), MPO (myeloperoxidase), MMP (matrix metalloproteinase), ApoA-1 (apolipoprotein A-1), ApoB-100

(apolipoprotein B-100), LDL (low-density lipoprotein), LYVE-1 (lymphatic vessel endothelial hyaluronic acid

receptor-1), Prox1 (prospero-related homeobox 1).

** Novus Biologicals, Littleton, CO, USA; Dako, Glostrup, Denmark; Abcam, Amsterdam, Netherlands; Thermo Fisher

Scientific, Carlsbad, CA, USA; Cell Marque, Rocklin, CA, USA; Sigma-Aldrich, St. Louis, MO, USA; Serotec, Oxford,

UK; Novocastra, Newcastle upon Tyne, UK; Monosan, Uden, The Netherlands; R&D Systems, Minneapolis, MN,

USA.

IHC: immunohistochemical staining

IF: immunofluorescence staining

(I) used in this work.

(II) used in previous work: ^3^

(III) used in previous work: ^4^

(IV) used in previous work: ^5^

(V) used in previous work: ^6^

(VI) used in previous work: ^7^

1. Young SG, Witztum JL, Casal DC, Curtiss LK, Bernstein S. Conservation of the low density lipoprotein receptor-binding domain of apoprotein B. Demonstration by a new monoclonal antibody, MB47. *Arterioscler Dallas Tex*. 1986;6(2):178-188. doi:10.1161/01.atv.6.2.178

2. Palinski W, Ylä-Herttuala S, Rosenfeld ME, et al. Antisera and monoclonal antibodies specific for epitopes generated during oxidative modification of low density lipoprotein. *Arterioscler Dallas Tex*. 1990;10(3):325-335. doi:10.1161/01.atv.10.3.325

3. Ollikainen E, Tulamo R, Frösen J, et al. Mast cells, neovascularization, and microhemorrhages are associated with saccular intracranial artery aneurysm wall remodeling. *J Neuropathol Exp Neurol*. 2014;73(9):855-864. doi:10.1097/NEN.0000000000000105

4. Huuska N, Netti E, Tulamo R, et al. Serum Amyloid A Is Present in Human Saccular Intracranial Aneurysm Walls and Associates With Aneurysm Rupture. *J Neuropathol Exp Neurol*. 2021;80(10):966-974. doi:10.1093/jnen/nlab086

5. Ollikainen E, Tulamo R, Lehti S, et al. Myeloperoxidase associates with degenerative remodeling and rupture of the saccular intracranial aneurysm wall. *J Neuropathol Exp Neurol*. 2018;77(6):461-468. doi:10.1093/jnen/nly028

6. Ollikainen E, Tulamo R, Lehti S, et al. Smooth Muscle Cell Foam Cell Formation, Apolipoproteins, and ABCA1 in Intracranial Aneurysms: Implications for Lipid Accumulation as a Promoter of Aneurysm Wall Rupture. *J Neuropathol Exp Neurol*. 2016;75(7):689-699. doi:10.1093/jnen/nlw041

7. Huuska N, Netti E, Lehti S, Kovanen PT, Niemelä M, Tulamo R. Lymphatic vessels are present in human saccular intracranial aneurysms. *Acta Neuropathol Commun*. 2022;10(1):130. doi:10.1186/s40478-022-01430-8
