# Supplementary figures and images for: CD4+ T-lymphocytes in human saccular intracranial aneurysm walls are associated with aneurysm rupture
Source: J Neuropathol Exp Neurol. 2025 Jun 11;84(10):870–8. doi: 10.1093/jnen/nlaf060 (PMC12456882; doi:10.1093/jnen/nlaf060)

## Slide 1
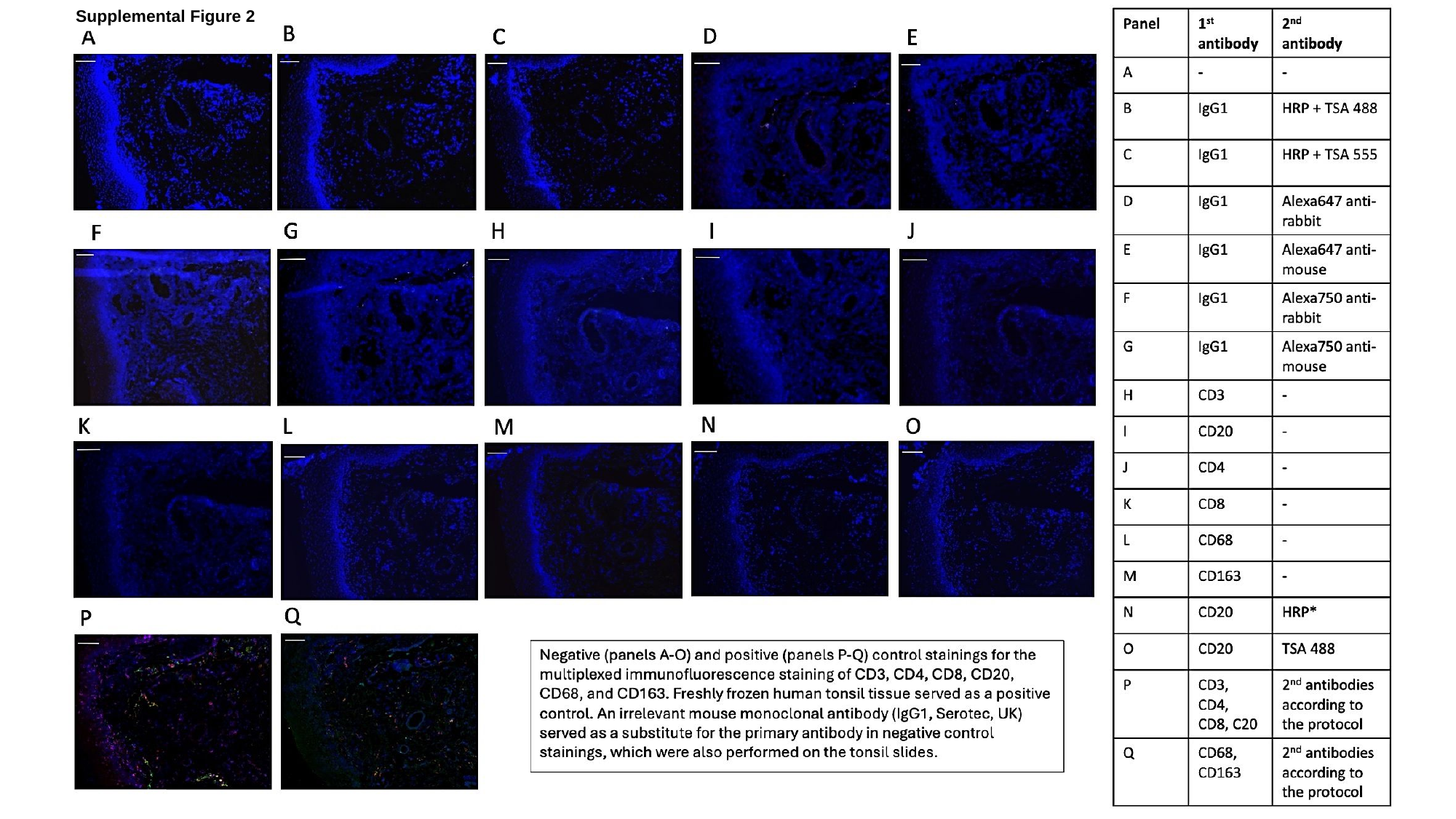

Supplemental Figure 2

Supplement: nlaf060_Supplementary_Data [file nlaf060_supplementary_data.zip › Supplementary Data/Figure S2.pptx]
